# Who Is at High Risk for Child Abuse and Neglect: Risk Assessment among Battered Women Using Shelter Services

**DOI:** 10.3390/ijerph20010833

**Published:** 2023-01-01

**Authors:** Ko Ling Chan, Mengtong Chen, Camilla K. M. Lo, Xiao Yan Chen, Debbie Tang, Patrick Ip

**Affiliations:** 1 Department of Applied Social Sciences, The Hong Kong Polytechnic University, Hung Hom, Hong Kong, China; 2 Department of Social Work, The Chinese University of Hong Kong, Shatin, Hong Kong, China; 3 Family Crisis Support, Po Leung Kuk, Causeway Bay, Hong Kong, China; 4 Department of Paediatrics and Adolescent Medicine, The University of Hong Kong, Pokfulam, Hong Kong, China

**Keywords:** refuge center, battered women, co-occurrence, child abuse and neglect, intervention plan

## Abstract

Background: The intersections between intimate partner violence (IPV) and child abuse and neglect (CAN) have received growing attention from the research community. However, there is limited research examining the risk factors for CAN among children of battered women who have experienced severe IPV and seek refuge in shelters. Objective: In the current study, we examined the co-occurrence of IPV and CAN and the risk factors for CAN in a sample of battered women. Participants and Setting: We recruited 260 battered women who were staying in women’s shelters in Hong Kong. Methods: We analyzed the data collected from the risk assessment reports of battered women and focused on IPV against women, CAN, and risk assessment. Results: Nearly half of the battered women had reported both IPV against themselves and CAN against their children. These women were, in general, younger, unemployed, and had been living in Hong Kong for less than seven years as new immigrants. Other risk factors for CAN in violent families included women’s conflicts with their partner and abusers with higher levels of stress and approval of violence. Conclusions: This exploratory study of risk factors for the co-occurrence of IPV and CAN advances our understanding of the causes of violence against women and children in families with violence. Our findings suggest that additional integrated services should be offered to both battered women and their children during their stay in shelters and after shelter departure. Addressing IPV and CAN and reducing adverse consequences needs greater collaboration among the various stakeholders across the social services, health, educational, and legal sectors.

## 1. Introduction

Violence against women and children is recognized as a global public health problem and human rights violation [1]. Women and children are often victims of family violence and the intersections between intimate partner violence (IPV) and child abuse and neglect (CAN) have received growing attention in the literature. According to the World Health Organization, IPV is “one of the most common forms of violence against women and includes physical, sexual, and emotional abuse and controlling behaviors by an intimate partner” [2], and CAN refers to physical, psychological, and sexual abuse and neglect “at the hands of parents and other authority figures” [3]. Considerable research has used community samples to investigate the prevalence of the co-occurrence of IPV and CAN, their shared risk factors and underlying social norms, and the resulting health consequences for women and children [4,5,6]. CAN is more prevalent in families identified with severe IPV in which women are seeking refuge in emergency shelters than it is in other families [7]. Severe IPV can prompt battered women to flee from their violent partners, and they often bring their children along [8].

Children exposed to IPV can be considered victims of CAN, as the environment they are living in is psychologically abusive and neglectful, and physical and sexual abuse may be occurring [9]. Witnessing parental domestic violence can increase the risk of exposure to violence and a variety of negative health outcomes in later life [10]. Efforts seek to prevent multiple forms of violence within families by addressing the shared factors of IPV and CAN [1]. Identification of and screening for these risk factors in families with violence will be essential to prevent severe CAN. Refuge centers tend to capture some of the most extreme cases of IPV and CAN. Thus, by using the risk assessments for battered women using refugee services in Hong Kong, the current study sought to contribute to the existing literature by exploring the factors that are associated with the risks for CAN in families with violence.

### 1.1. The Intersection of IPV and CAN: High Co-Occurrence and Severe Consequences

Substantial evidence confirms that IPV and CAN co-occur. For example, exposure to childhood abuse and trauma is associated with an increased risk of IPV exposure in adulthood [11,12,13,14,15]. The association is often explained by the theory of intergenerational transmission of violence [16]. Growing attention has adopted a holistic approach to assessing families with violence and investigating the overlap between IPV and CAN in the same families [5]. Although the rate of overlap varies across different studies due to different sample populations, assessment methods, and cultures, the association between CAN and IPV remains consistent [6]. An earlier review on the intersection of spousal abuse and child physical abuse showed that the co-occurrence rates in community samples was approximately 6% and ranged from 20% to 100% in clinical samples [17]. According to a recent meta-analysis of co-occurrences of family violence, the co-occurrence rate of IPV and CAN was 9% in community samples and 38.6% in clinical samples of either battered women or abused children [4]. Existing studies have mostly focused on CAN by the IPV perpetrator, with the co-occurrence rate ranging from 11–97% [7]. However, battered women can also become perpetrators of CAN [18,19]. 

In a population-based sample in Hong Kong, 54.4% of the families with the occurrence of IPV were involved in child physical abuse over the child’s lifetime, and 46.5% were involved in child physical abuse in the preceding year [20]. A large-scale study of a representative student sample in Hong Kong reported that the lifetime co-occurrence rate of the two types of family violence was 12.3%, and was 3.6% for a co-occurrence during the preceding year [21]. The figures show that children living in families with violence identified in community surveys are at risk of abuse and neglect, and research has suggested that children living in families in which women are seeking refuge from IPV at a shelter are at greater risk of abuse and neglect than other children [4,7].

A range of adverse health and social consequences for victims of IPV or childhood maltreatment have been well documented in existing studies [22,23]. Although the overlap of CAN and children’s exposure to IPV makes it difficult to determine whether the consequences are distinguishable, evidence has shown that the effects on children who were dually exposed are compounding and highly negative [6,24]. Studies have also examined the impact that cumulative exposure to abuse has on women, but most of those studies have focused only on physical or sexual abuse in the victims’ childhood and their adulthood IPV [25,26]. Based on a sample of women who were admitted to domestic violence shelters with over half of their children being direct victims of abuse, Fernández-González et al. [27] found that up to 80% of women had mental health issues. 

### 1.2. Risk Factors for Co-Occurrence of IPV and CAN

Different forms of violence within the family often stem from the same factors [28]. Within an ecological framework, risk factors related to individual, family, community, and societal characteristics can contribute to the co-occurrence of IPV and CAN in the same families [29]. To date, research has emphasized assessing the risks posed by the perpetrator. A range of perpetrator-related factors that increase the risks of IPV and CAN have been identified, such as obsessive controlling behavior, attitudes of acceptance of IPV and CAN, substance misuse, crime, and mental health problems [30,31]. In addition, women who are pregnant, are isolated from social support, or have been sexually assaulted are more likely than others to become the victims of domestic violence [31] and can even perpetrate CAN [32]. Marital conflict can significantly increase the risk of using harsh discipline in parenting [33]. In addition to the perpetrators of IPV, the victims are also likely to use negative parenting practices, including physical aggression and neglect [18]. Family factors associated with the co-occurrence of IPV and CAN include poverty, unemployment, parenting stress, and poor relationship quality and communication [6]. The co-occurrence can be explained through the spillover effect, which posits that in a family with inadequate resources to resolve a relationship conflict, the adverse effect of that relationship can spill over onto another familial relationship [29]. In addition, neighborhood characteristics, such as low socioeconomic status, high levels of policing activity, and residential instability were also found to be associated with higher-than-average risks of IPV and CAN [34]. 

Although several plausible explanations exist for the co-occurrence of IPV and CAN, due to the range of risk factors identified using nonviolent families as the comparison group, the within-group differences between different conditions of IPV or CAN in families with violence are still unclear. Research has shown that female survivors of IPV at shelters are at an increased risk for CAN [35,36]. Anderson et al. [37] found that IPV-related PTSD symptoms among women in shelters were strongly related with child abuse potential. However, a limitation in the literature has been the lack of comparisons between families with only IPV and those with both IPV and CAN. A study further examining the whole risk profile of the family with violence is needed and could provide clinical implications for the assessment of CAN and the provision of services for battered women and children.

### 1.3. The Current Study

The co-occurrence of IPV and CAN is considerably more prevalent in clinical samples than in population studies, although those at high risk for both IPV and CAN have not been identified. In this exploratory study, by working with a sample of battered women seeking refuge in shelters in Hong Kong, we examined the degree of overlap between IPV and CAN and the associations between victimization experiences and the health consequences of women and their children. We sought to evaluate the risk profile of the battered women and examine the extent to which their demographic characteristics, socioeconomic status, relationship factors, health status, and victimization experiences are associated with CAN in the family with violence. Finally, we have summarized the current intervention plans for battered women and provide recommendations to bridge the gaps in current services for battered women and their children. 

## 2. Materials and Methods

### 2.1. Research Site and Sample

This study used data collected from three major refuge centers for battered women in Hong Kong that are under the operation of Po Leung Kuk, a local charitable organization founded as the society for the protection of women and children. In addition to providing temporary accommodations, the centers conduct risk assessments and provide professional services to help women victims handle emotional distress, establish safety plans, and minimize the negative impacts of domestic violence on their children. We obtained the risk assessment records of 260 women living in the refuge centers in the years 2020–2021. The sample was not limited to battered women who have dependent children, but 87% of them reported that they had children. Ethical approval was granted by the Ethics Committee of a university in Hong Kong.

### 2.2. Measures 

#### 2.2.1. Demographic Characteristics

The battered women participants were asked to provide demographic information about themselves, their abusers, and their children, including their relationship with their abusers, their co-residence status (yes or no), ethnicity (Chinese or ethnic minorities), age, gender (male or female), years lived in Hong Kong, educational level (primary and below, secondary, tertiary and above), employment (yes or no), disabilities (yes or no), mental health status (yes or no), marital status (married, cohabiting, divorced/separated, others), number of children, and whether they were pregnant (yes or no). 

#### 2.2.2. IPV against Women

The female respondents reported the specifics of their IPV victimization by their current partner using the Chinese version of the Revised Conflict Tactics Scale (CTS2; [20]). A total of 33 items were used in four subscales: physical assault (e.g., throwing something at me, twisting my arm or hair, pushing or shoving, etc.), psychological aggression (e.g., insulting and swearing, shouting or yelling, saying something spiteful, etc.), sexual violence (using force to make me have sex, insisting on sex without a condom, etc.), and injury (cut or bleeding, having a sprain or bruise, etc.). The items were designed using a four-point scale (0 = never happened, 1 = happened in the recent 6 months, 2 = happened in the recent 7–12 month, 3 = happened before the past 12 months). The CTS2 showed satisfactory reliability in this study (α = 0.77–0.87).

#### 2.2.3. Child Abuse and Neglect

The Parent–Child Conflict Tactics Scale (CTSPC; [38]) was used to obtain information on CAN in terms of its prevalence over their lifetime and during the preceding year, and the types of abuses, including physical assault (e.g., pushing, shoving, grabbing, slapping, kicking, chocking, etc.), psychological aggression (e.g., yelling, insulting, swearing, threatening, etc.), sexual coercion (e.g., touching a child’s private parts, taking pictures or video of a child’s nude body that were against the child’s will), and neglect (e.g., not getting enough food, not being taken to the doctor when sick, or not having a safe living environment). The battered women responded to the items about whether their children had experienced each type of aggression above (1 = yes, 0 = no). The psychometric characteristics of the instrument, including its reliability and discriminant and construct validity, have been well documented [38]. The CTSPC was translated into Chinese using the back-translation method and has been validated and demonstrated to have satisfactory reliability [39].

#### 2.2.4. Risk Assessment

To identify the risk levels of the women and children in the refuge centers, social workers and clinical psychologists were trained to first conduct a standardized risk assessment using a checklist that had been previously validated [40]. The assessment focused on the context of the victimizations, including the duration of being victimized, the types and severity of the injuries, the acts of harm by the abusers, the threats faced by the women, the physical and mental health status of the women, and whether the women had suicidal ideation or behavior.

The risk factors and protective factors for IPV were also assessed. These risk factors included the health status of battered women/family members/abusers, their socioeconomic status, intimate-partner relationships (e.g., having frequent daily conflict with partner, considerable spousal age difference, etc.), indebtedness, addiction to gambling, experiences of childhood maltreatment, recidivism, emotional or behavioral issues, alcohol or substance abuse, antisocial personality disorder, borderline personality disorder, and so on. The protective factors for IPV included parent–child relationships, parenting efficacy, parenting-stress coping style, social networks, and support from extended family members.

On the basis of the above information assessed by the social workers and clinical psychologists, the risk level for the severity of violence, the risk of recidivism, and the overall level of risk were next rated as low, medium, or high. Though the assessment may be subjective when compared with an actuarial risk assessment, the clinical risk assessment is more practical and clinically reliable for informing risk management. The interrater reliability was tested, and it maintained over 98% agreement. The assessors were also required to establish a safety plan and an immediate intervention plan.

### 2.3. Statistical Analysis

Descriptive analyses were performed for background characteristics and victimization experiences of the battered women and their children. Chi-square tests and t-tests were performed to compare the differences between women who experienced only IPV and those who experienced both IPV and CAN. Odds ratios (ORs) were calculated to explore the factors that were associated with increased and decreased likelihoods that IPV and CAN would co-occur. For the variables related to child victims, we chose the oldest child reported. The missing data were excluded listwise in the analyses.

## 3. Results

Table 1 shows the demographic characteristics of the battered women. Among the 260 participants, 127 (48.85%) had experienced both IPV and CAN. Their mean age was 40.6 years (SD = 11.4). More than 90% of the participants were Chinese; more than 90% of the participants were in a marriage or cohabiting relationship. Only 15% of the women had a tertiary level education, half of them had a monthly income lower than HKD 7000 (about USD 900), and nearly 20% received Comprehensive Social Security Assistant (CSSA). Nearly 20% of the battered women had been diagnosed with mental illness.

Table 2 shows the demographic characteristics of the children under 18 years of age who were living in the refuge centers with their mothers. The mean age of the children was 7.39 years (SD = 4.62). There were slightly more boys than girls. More than 70% of the children had been born in Hong Kong. A small percentage of the children had chronic health conditions or had been diagnosed with a mental illness.

Among the 289 children, 186 (64.4%) had experienced CAN. The children who were victims of CAN (mean age = 7.82 years, SD = 4.62) were older than those who were non-victims (mean age = 6.62, SD = 4.54). When we computed the women’s sample, 133 (51.15%) of the women had reported only IPV and 127 (48.85%) of the women had reported both IPV against themselves and CAN against their children (Table 1). The women in the IPV and CAN co-occurrence group were significantly younger than the other women, more were new immigrants, more were unemployed, but fewer had chronic health conditions. 

### 3.1. Victimization Experiences and Health Outcomes of Battered Women

Table 3 summarizes the victimization experiences of the battered women at the refuge centers. The vast majority of the abusers—95.5%—were the partners of the battered women, and only 4.5% of the abusers were the women’s ex-partners. Half (52.8%) of the women reported they had been victims of IPV for less than five years, 22.3% reported having been victimized for 5–10 years, and 24.9% reported more than 10 years of abuse. In terms of the abusive incidents, 68.9% of the women had experienced 1–5 times of IPV, 12% had experienced 6–10 times of IPV, and 19.1% had experienced more than 10 times of IPV. More than half of the participants agreed that, compared with previous years, the past-year victimization was more frequent. More than 40% of the women had an injury or other physical issues at the time of the first interview, and 18.5% had a severe or permanent injury. In the preceding year 76.4% of the women had been threatened, and 76.7% were afraid of their abuser. Roughly 20% of the women were harassed by their abuser, and 57.7% believed the abuser would track them or their children down. Women in the IPV and CAN co-occurrence group reported a higher number of abusive incidents and forced sex by the abuser in the previous year than the other women did. 

Most of the women participants reported health problems resulting from their abuse. A total of 65% of the women had one or more health issues, such as insomnia (37.7%), feeling upset (31.2%), feeling panic/fear/nervousness (27.3%), and having headaches (20.0%). More than half of the women had suicidal ideation. 

### 3.2. Victimization Experiences of the Children Living in Shelters

Table 4 shows that among the children who had been abused or neglected, the rates of psychological aggression, physical assault, neglect, and sexual abuse were 90.3%, 62.9%, 10.2%, and 1.1%, respectively. Most of the physical and psychological abuse and neglect against the children had occurred in the previous six months and was committed by the perpetrator of IPV, with the rates ranging from 62.4% to 83.3%. Considerable percentages of children also had experienced abuse and neglect perpetrated by the battered women, with the rates ranging from 11.9% to 17.1%.

### 3.3. Risk Factors of CAN among Battered Women

Table 5 shows the risk factor correlates for CAN. The analysis focused mainly on the characteristics that were likely to be associated with CAN among IPV victims. The associations with CAN appeared to be stronger if the battered mother was a new immigrant to Hong Kong (OR = 1.78, *p* < 0.5), young (OR = 0.94, *p* < 0.001), unemployed (OR = 1.72, *p* < 0.05), or had frequent conflicts with her partner (OR = 4.58, *p* < 0.01). The abusers who committed IPV, who had a relatively higher level of approval of violence (OR = 2.39, *p* < 0.1), and a relatively higher level of stress (OR = 2.59, *p* < 0.1) were more likely to also perpetrate CAN.

### 3.4. Risk Assessment and Management

**Figure 1:**
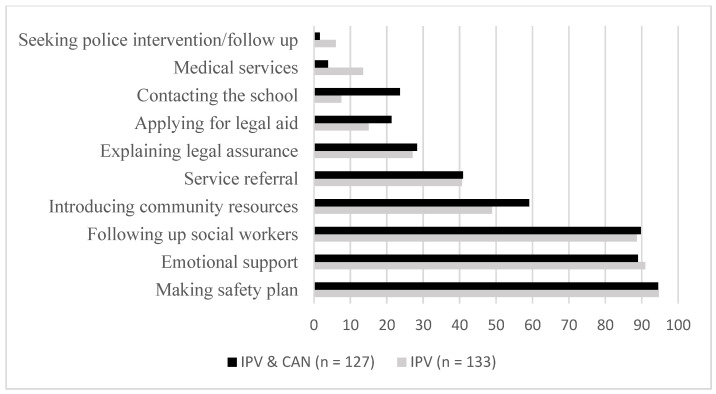
Intervention plan for battered women and children.

Table 6 presents the results of the risk assessments conducted by social workers and clinical psychologists and shows how the occurrences were distributed among the three risk levels, for women with only IPV versus with both IPV and CAN. More than 80% of the battered women had experienced severe violence, and 74.9% faced a higher risk of recidivism. In general, more than 85% of the women living in the refuge centers were at high risk for fatal or serious harm in the future. Specific intervention plans based on the risk assessment were made by social workers for the women living in the refuge centers. As Figure 1 shows, the most common action plans included making safety plans, providing emotional support, and following up with a social worker. Those plans were followed by introductions to community resources and by service referrals and legal assistance. A small percentage of the abused women needed assistance in applying for legal aid, contacting their child’s school, utilizing medical services, and seeking police intervention. Women who experienced only IPV received more medical services than did those who experienced both IPV and CAN, whereas the women who experienced both IPV and CAN were introduced to more community resources and received help contacting their children’s schools. 

## 4. Discussion

IPV is known to be a major risk factor that is associated with CAN. In addition, the co-occurrence rates of IPV and CAN are much higher in families with severe partner violence. In this study examining a clinical sample, almost half of the battered women reported that their children were victims of CAN—that is, 64.4% of children whose mothers sought refuge in a shelter had experienced CAN. Older children were more likely to become victims of CAN when their battered mothers were younger, had been living in Hong Kong for less than seven years as new immigrants, and were unemployed. This profile of battered women reflects, to some extent, the cross-border marriage phenomenon in Hong Kong, where marriage migrants are generally younger, more likely to be unemployed, and often have low socioeconomic status, thus displaying significantly different characteristics from local women [41]. As of 2016, the proportion of Hong Kong–Mainland China cross-border marriages accounted for 34.7% of marriages registered in Hong Kong, which has increased significantly from the pre-handover period [42]. These women are often more socially isolated and have less social support [41]. Thus, marriage migration may involve these certain intrinsic risk factors for IPV victimization [43]. Interestingly, despite significant differences among battered women with different violent experiences, it should be noted that in general most had low levels of education and income, and the majority were still in a marital/cohabiting relationship.

In this study, the battered women who also reported CAN had experienced a much higher frequency of partner violence in the previous year than the other women had. In addition, in the previous year a majority of them had been forced by their partners to have sex. Proportionately more battered women with CAN experiences believed their abusers would track them and their children down. However, it should be noted that the experiences of IPV, in terms of the years of being victimized, the frequency of violence in the previous year, the severity of the IPV, the experience of being threatened, and their attitudes toward their abusers, were generally similar for all of the battered women, regardless of whether they also reported CAN. The findings thus indicate that even children who were not victims of direct abuse and neglect may have witnessed severe violence against their mother. Consistently with previous studies, battered women can also become perpetrators of CAN, although the majority of the perpetrators of CAN were the women’s partners [18,32].

A number of risk factors were identified among the battered women, the abusers, and other family members. A high percentage of women reported that their families had low socioeconomic status, including receiving Comprehensive Social Security Assistance (CSSA), and a majority were new immigrants and unemployed. Mental illness and chronic diseases were common in families with violence. However, those with chronic diseases were less likely than others to perpetrate CAN. Daily conflict with the partner was a very common risk factor and was more prevalent in families with CAN. Very common risk factors of abusers included a weak ability to control anger, being keen on face-saving, shirking responsibilities, manipulating their partner, experiencing stress, and approval of violence. In particular, high levels of approval of violence and facing strong pressure were correlated for CAN among the abusers of intimate female partners. It should be noted that, in the current study, even though several factors were not found to be significantly associated with the co-occurrence of IPV and CAN in families with violence, they still could significantly increase the risks compared to nonviolent families.

### 4.1. Implications 

This study’s assessment of battered women suggests that more than 85% of these women are at high risk and have complex situations and thus require comprehensive services that address a range of different needs of both the women and their children. It is important to promote assessment of risks and needs of women and children who seek refuge in a shelter after IPV victimization. During battered women’s short-term stays in refuge centers, social workers can use the results of their risk assessments to help the victims set up safety plans and assist them in resolving their situations, with the aim of restoring their autonomy in life. Professional services should be provided to help battered women handle emotional distress. In addition, the refuge centers should provide child services to help children adapt to the changes in their life. In consideration of the negative impacts on the children of witnessing IPV, the refuge centers should also provide interventions to minimize the impact of domestic violence on the children [44], including preventing the intergenerational transmission of violence in the future.

This study’s findings have implications for practices to bridge some of the gaps in the services. First, the high co-occurrence of IPV and CAN among battered women, along with the complex links identified in this study, suggest the profound importance of treatment for CAN in the context of families with violence. In addition to services to help children living in the shelters adapt to the changes in their lives, additional integrated services should be offered to them because a very high proportion of such children have been abused or neglected. In addition, specialized services should be provided for children who witness IPV only, are victims of physical or psychological abuse, or are sexually abused [45]. For example, tertiary prevention services provided by professional social workers and mental health counselors are needed to reduce the adverse health consequences for children of IPV families in refuge centers [6,24].

Second, interventions addressing the multiple shared factors that link IPV and CAN should contribute to preventing both forms of violence [1]. This could be both effective and cost-effective. Although an understanding of the impacts of perpetrator risk factors is essential, the perpetrator risk assessment may not provide an easy fix for IPV or CAN prevention [31]. It will be important to make additional resources available to battered women, especially for those who are young, unemployed immigrants, so that they can restore their autonomy in life. In addition, access to safe and affordable childcare is vital for battered women with children who are exposed to violence.

Third, domestic violence can have long-term consequences both for battered women and their children [6]. Follow-up interventions after the immediate intervention at refuge centers are needed. Further services must also be provided to battered women after they depart the shelters [7]. For example, home visitation interventions can also be effective in preventing further CAN by teaching mothers to implement appropriate parenting practices and by providing them with instrumental and emotional support [46]. In addition, it will be essential to create pathways from temporary shelter centers to long-term safe housing for the battered women and their children escaping a violent partner.

Fourth, addressing multiple forms of violence and reducing the adverse consequences of that violence will require greater collaboration among social workers, health professionals, childcare providers, teachers and other school personnel, and law enforcement officers. Additional efforts are needed to eliminate any remaining barriers to safety and justice for all victims. For example, police officers in Hong Kong are likely to adopt a non-intervention approach when handling domestic violence, and that narrow approach reflects a need to train the police in risk assessment and holistic thinking about human rights [47]. In addition, the mainstream support services for battered women and their children target primarily Chinese-speaking or English-speaking clients, and information about support services and actual services should also be made available to ethnic minorities in Hong Kong [48].

### 4.2. Limitations

We also acknowledge the study’s limitations. First, this study was based on the reports from battered women who sought refuge from IPV at shelters in Hong Kong. However, the study did not include some hard-to-reach populations who had experienced severe violence but were not willing to disclose that fact. Thus, the generalizability of this study to higher-risk samples may be limited. Second, CAN was assessed with self-reported measures, i.e., the mothers of abused children, which implies the incidence may be underestimated. Third, because this study was cross-sectional, causal relationships between the correlates and the co-occurrence of IPV and CAN cannot be drawn. Fourth, as we have performed multiple univariate logistic regressions to examine several factors associated with the cooccurrence of IPV and CAN, the risk for Type 1 error may increase.

Furthermore, whereas the evidence about the intersections of IPV and CAN has grown in recent years, knowledge about the profiles of children who enter refuge centers together with their battered mothers is still insufficient, including their risks for CAN. Thus, services responding to the intersections of IPV and CAN are still preliminary. It will be important for future studies to explore the risk factors for CAN in families with violence by using larger and more diverse samples, including those non-help-seeking families. These samples may help explain the variability in the risks of co-occurrences of IPV and CAN. Moreover, there is a need for piloting intervention programs to reduce the negative impacts of IPV and CAN both on the children and their battered mothers. Evidence of the effectiveness and cost-effectiveness of interventions should inform future practices, including enhancing collaborations among social workers, health professionals, teachers, and law enforcement officers.

## 5. Conclusions

Based on a clinical sample of battered women living in refuge centers in Hong Kong, we found high co-occurrence rates of IPV and CAN in families with severe partner violence. We further examined the risk factors for the co-occurrence of IPV and CAN in families with severe violence. It is important to minimize the impact of domestic violence on battered women and their children that additional integrated services should be offered during their stay in shelters and after shelter departure. Greater collaboration among the various stakeholders across the social services, health, educational, and legal sectors is needed.

## Figures and Tables

**Table 1:** Demographic Characteristics of the Battered Women in the Refuge Centers.

Characteristics	Total (*N* = 260)		IPV Only (*N* = 133)		IPV and CAN (*N* = 127)		χ^2^/*t*
	*n*	%	*n*	%	*n*	%	
Age range, in years							41.75 ***
18–30	45	17.4	23	17.3	22	17.5	
31–40	102	39.4	35	26.3	67	53.2	
41–50	69	26.6	35	26.3	34	27.0	
>50	43	16.6	40	30.1	3	2.4	
Age: Mean, SD	40.6	11.4	43.8	13.8	37.2	6.9	4.95 ***
Ethnicity							0.11
Chinese	238	91.5	121	91.0	117	92.1	
Non-Chinese	22	8.5	12	9	10	8.9	
No. of children: Mean, SD	1.7	0.75	1.68	0.69	1.74	0.83	−0.58
Residence							12.82 ***
Born in HK	68	26.2	43	32.3	25	19.7	
<7 years in HK (new immigrants)	84	32.3	30	22.5	54	42.5	
7 years or longer	99	38.1	55	41.4	44	34.6	
Non-local	9	3.5	5	3.8	4	3.1	
Marital status							0.04
Married/Cohabiting	213	91.4	101	91.8	112	91.1	
Divorced/Separated/Broken up	20	8.6	9	8.2	11	8.9	
Educational attainment							2.02
Primary or below	44	17.0	26	19.7	18	14.2	
Secondary	176	68.0	89	67.4	87	68.5	
Tertiary or above	39	15.0	17	12.9	22	17.3	
Monthly income							1.19
Less than HKD 7000	70	50	34	48.6	36	51.4	
HKD 7000 to HKD 14,999	57	40.7	31	44.3	26	37.1	
HKD 15,000 or above	13	9.3	5	7.1	8	11.4	
Receiving CSSA	46	17.7	18	13.5	28	22.0	3.23
Unemployed	155	59.6	71	53.4	84	66.1	4.46 *
Has chronic health conditions	68	26.2	49	36.8	19	15.0	16.11 ***
Diagnosed with mental illness	50	19.2	29	21.8	21	16.5	1.16

Note. IPV = intimate partner violence; CAN = child abuse and neglect; CSSA = Comprehensive Social Security Assistance. *** *p* < 0.001, * *p* < 0.05.

**Table 2:** Demographic Characteristics of the Children in the Refuge Centers.

Characteristics	Total (*N* = 289)		IPV Only (*N* = 103)		IPV & CAN (*N* = 186)		χ^2^/t
	*n*	%	*n*	%	*n*	%	
Age range, in years							3.52
0–5	114	39.4	48	46.6	66	35.5	
6–11	111	38.4	34	33.0	77	41.4	
12–17	64	22.1	21	20.4	43	23.1	
Age: mean, SD	7.39	4.62	6.62	4.54	7.82	4.62	−2.11 *
Gender							0.88
Male	151	52.2	50	48.5	101	54.3	
Female	138	47.8	53	51.5	85	45.7	
Residence							1.7
Born in HK	205	70.9	76	73.8	129	69.4	
<7 years in HK (new immigrants)	65	22.5	19	18.5	46	24.7	
7 years and longer	12	4.2	5	4.9	7	3.8	
Non-local	7	2.4	3	2.9	4	2.2	
Has chronic health conditions	11	3.8	2	1.9	9	4.8	1.52
Diagnosed with mental illness	11	3.8	3	2.9	8	4.3	0.35

Note. IPV = intimate partner violence, CAN = child abuse and neglect. * *p* < 0.05.

**Table 3:** IPV Victimization and Health Outcomes of Women Participants.

Victimization/Outcome	Total (*N* = 260)		IPV Only (*N* = 133)		IPV & CAN (*N* = 127)		χ^2^/t
	*n*	%	*n*	%	*n*	%	
Abuser (harmed or threatened to harm participant)							0.54
Partner	210	95.5	101	94.4	109	96.5	
Ex-partner	10	4.5	6	5.6	4	3.5	
Years of being victimized							4.95
<5 years	123	52.8	66	55	57	50.4	
5–10 years	52	22.3	20	16.7	32	28.3	
>10 years	58	24.9	34	28.3	24	21.2	
No. of abusive incidents in the past year							3.41
1–5 times	126	68.9	69	74.2	57	63.3	
6–10 times	22	12.0	11	11.8	11	12.2	
>10 times	35	19.1	13	14.0	22	24.4	
No. of abusive incidents in the past year: Mean, SD	11.9	35.4	6.9	20.8	17.4	45.9	−2.13 *
Compared with previous years, the past-year’s victimization was							3.5
More frequent	123	53.9	63	53.8	60	54.1	
Comparable	71	31.1	41	35.0	30	27.0	
Less frequent	34	14.9	13	11.1	21	18.9	
Currently has injuries or other physical issues	97	40.6	57	45.6	40	35.1	2.73
Level of injury							0.58
Minor (e.g., scratches, bruises, redness)	110	81.5	62	83.8	48	78.7	
Severe and/or permanent (e.g., fractures, severe bruises, coma, blindness, hearing loss, etc.)	25	18.5	12	16.2	13	21.3	
Has been threatened by the abuser in the past year	198	76.4	97	73.5	101	79.5	1.31
Has been forced to have sex with the abuser in the past year	64	24.9	24	18.3	40	31.7	6.19 *
Is afraid of the abuser	197	76.7	98	74.8	99	78.6	0.51
Is still in contact with the abuser	74	28.9	37	28.0	37	29.8	0.10
Thinks the abuser will track her and her children down	142	57.7	64	41.6	78	63.9	3.83 ^#^
Is still currently harassed by the abuser	52	20.5	25	18.9	27	22.1	0.4
Current health problems							
Insomnia	98	37.7	53	39.8	45	35.4	0.61
Headache	52	20.0	28	21.1	24	18.9	0.20
Muscle pain	23	8.8	10	7.5	13	10.2	0.60
Loss of appetite	34	13.1	19	14.3	15	11.8	0.37
Tiredness	42	16.2	17	12.8	25	19.7	2.33
Substance dependence	10	3.8	7	5.3	3	2.4	1.50
Panic/Fear/Nervousness	71	27.3	36	27.1	35	27.6	0.01
Upset	81	31.2	51	38.3	30	23.6	7.09
Anxious	29	11.2	16	12.0	13	10.2	0.22
Other	35	13.5	21	15.8	14	11.0	1.31
Any symptom above	169	65.0	90	67.7	79	62.2	0.85
Has suicidal ideation	142	54.6	77	57.9	65	51.2	1.18

Note. IPV = intimate partner violence, CAN = child abuse and neglect. * *p* < 0.05, ^#^ *p* = 0.05.

**Table 4:** Abuse and Neglect against Children.

Forms of Abuse/Neglect	IPV & CAN (*N* ^ = 186)		Perpetrator–Women						Perpetrator–Partner					
	*n*	%	*n*	Past 6 Months (%)		Past 12 Months (%)	*n*	Before 12 Months (%)	*n*	Past 6 Months (%)	*n*	Past 12 Months (%)	*n*	Before 12 Months (%)
Psychological aggression	168	90.3	20	11.9	1	0.6	6	3.6	140	83.3	8	4.8	2	1.2
Physical assault	117	62.9	20	17.1	4	3.4	5	4.3	73	62.4	8	6.8	13	11.1
Sexual abuse	2	1.1	0	0	0	0	0	0		0	1	50.0	1	50.0
Neglect	19	10.2	3	15.8	1	5.3	0	0	14	73.7	2	10.5	1	5.3

Note. IPV = intimate partner violence, CAN = child abuse and neglect. ^ the number of children.

**Table 5:** Risk factors for CAN.

Variables	IPV Only (*N* = 133)		IPV & CAN (*N* = 127)		Crude OR
	*n*	%	*n*	%	
**Characteristics of battered women**					
Age: mean, SD	43.8	13.8	37.2	6.9	0.94 *** (0.92, 0.97)
Educational attainment (ref: tertiary or above)	115	86.5	105	82.7	0.71 (0.36, 1.40)
Unemployed	71	53.4	84	66.1	1.72 * (1.04, 2.85)
Divorced/Separated (ref: married/cohabiting)	9	8.2	11	8.9	1.10 (0.44, 2.77)
**Characteristics of battered women/family members**					
Disabled	8	6.0	10	7.9	1.32 (0.51, 3.47)
Mentally ill	35	26.3	37	29.1	1.17 (0.67, 2.03)
Chronic disease	57	42.9	35	27.6	0.50 ** (0.30, 0.84)
New immigrants	49	36.8	65	51.2	1.78 * (1.08, 2.92)
Low-income or receiving social security	62	46.6	63	49.6	1.08 (0.66, 1.78)
In debt	38	28.6	38	29.9	1.14 (0.66, 1.99)
Addicted to gambling	30	22.6	24	18.9	0.79 (0.43, 1.46)
Unemployed	91	68.4	86	67.7	1.03 (0.60, 1.76)
Spousal age difference (>10 years)	29	21.8	40	31.5	1.52 (0.87, 2.67)
Frequent daily conflict with partner	104	78.2	119	93.7	4.58 ** (1.66, 12.63)
Conflict with in-laws	14	10.5	25	19.7	1.93 (0.95, 3.92)
**Characteristics of abusers**					
Experienced childhood abuse or neglect	23	17.3	24	18.9	1.19 (0.61, 2.30)
Witnessed violence between parents	18	13.5	27	21.3	1.66 (0.84, 3.30)
History of criminal offence	20	15.0	25	19.7	1.52 (0.78, 2.94)
History of sexual abuse	4	3.0	1	0.8	0.26 (0.03, 2.34)
History of perpetrating CAN/IPV	49	36.8	60	47.2	1.52 (0.90, 2.57)
Low self-esteem	51	38.3	51	40.2	1.24 (0.73, 2.09)
Approves of violence	71	53.4	85	66.9	2.39 ** (1.28, 4.46)
Poor anger management	116	87.2	118	92.9	1.45 (0.53, 3.95)
High level of stress	74	55.6	96	75.6	2.59 ** (1.43, 4.70)
Keen on face-saving	116	87.2	106	83.5	0.59 (0.26, 1.32)
Disregards social norms	48	36.1	48	37.8	0.97 (0.57, 1.64)
Jealousy issues	67	50.4	64	50.4	0.90 (0.54, 1.51)
Shifts responsibility	109	82.0	116	91.3	2.13 (0.92, 4.94)
Manipulates partner	80	60.2	89	70.1	1.27 (0.74, 2.17)
Has suicidal ideation	27	20.3	25	19.7	0.98 (0.53, 1.81)
Alcohol addiction	32	24.1	33	26.0	1.06 (0.61, 1.87)
Drug abuse	15	11.3	22	17.3	1.63 (0.80, 3.31)
Depression	32	24.1	29	22.8	0.91 (0.50, 1.64)
Antisocial personality disorder	12	9.0	9	7.1	0.76 (0.31, 1.88)
Borderline personality disorder	11	8.3	7	5.5	0.62 (0.23, 1.66)

Note. IPV = intimate partner violence, CAN = child abuse and neglect. OR = odds ratio. Ref = reference group. CSSA = Comprehensive Social Security Assistance. *** *p* < 0.001, ** *p* < 0.01, * *p* < 0.05.

**Table 6:** Levels of Risks Associated with IPV and CAN.

Risks	Total (*N* = 260)		IPV Only (*N* = 133)		IPV & CAN (*N* = 127)		χ^2^
	*n*	%	*n*	%	*n*	%	
Severe violence							0.93
Low	22	8.5	12	9.1	10	7.9	
Medium	26	10.0	11	8.3	15	11.8	
High	211	81.5	109	82.6	102	80.3	
Recidivism							3.42
Low	12	4.6	6	4.5	6	4.7	
Medium	53	20.5	33	25.0	20	15.7	
High	194	74.9	93	70.5	101	79.5	
General risk							1.7
Low	6	2.3	2	1.5	4	3.1	
Medium	32	12.4	19	14.4	13	10.2	
High	221	85.3	111	84.1	110	86.6	

## Data Availability

Not applicable.
